# Isothermal chemical denaturation assay for monitoring protein stability and inhibitor interactions

**DOI:** 10.1038/s41598-023-46720-w

**Published:** 2023-11-16

**Authors:** Randa Mahran, Niklas Vello, Anita Komulainen, Morteza Malakoutikhah, Harri Härmä, Kari Kopra

**Affiliations:** Department of Chemistry, University of Turku, Henrikinkatu 2, 20500 Turku, Finland

**Keywords:** Biochemistry, Biotechnology

## Abstract

Thermal shift assay (TSA) with altered temperature has been the most widely used method for monitoring protein stability for drug research. However, there is a pressing need for isothermal techniques as alternatives. This urgent demand arises from the limitations of TSA, which can sometimes provide misleading ranking of protein stability and fail to accurately reflect protein stability under physiological conditions. Although differential scanning fluorimetry has significantly improved throughput in comparison to differential scanning calorimetry and differential static light scattering throughput, all these methods exhibit moderate sensitivity. In contrast, current isothermal chemical denaturation (ICD) techniques may not offer the same throughput capabilities as TSA, but it provides more precise information about protein stability and interactions. Unfortunately, ICD also suffers from limited sensitivity, typically in micromolar range. We have developed a novel method to overcome these challenges, namely throughput and sensitivity. The novel Förster Resonance Energy Transfer (FRET)-Probe as an external probe is highly applicable to isothermal protein stability monitoring but also to conventional TSA. We have investigated ICD for multiple proteins with focus on KRAS^G12C^ with covalent inhibitors and three chemical denaturants performed at nanomolar protein concentration. Data showed corresponding inhibitor-induced stabilization of KRAS^G12C^ to those reported by nucleotide exchange assay.

## Introduction

Protein stability is critical to their correct function in cell, but also biologics and many in vitro assays utilizing the native protein structure and function. Protein stability is also fundamental for determination of optimum conditions for protein expression, purification, and storage^1^.Therefore, it is important to understand how various conditions such as solvent components and temperature affects the stability of different proteins.

Nowadays, thermal shift assays (TSAs) are the most used methods to assess protein stability^2^*.* This is due to simple TSA protocols, good throughput, and easy automation. These assays monitor protein stability during the temperature increase and can be used also in the presence of potential binding partners. Typically, all TSAs are based on fluorescence readout, in which the denaturation is seen as an increase in observed signal. On the other hand, protein–ligand interaction can be visualized as an increase in protein stability. In drug discovery, the shift in protein melting temperature (Δ* T*_m_) has become a popular parameter to identify potential protein binding ligands. However, the change in *T*_m_ values does not directly reflect the binding affinities or rank order of the ligands, as molecule binding to distinct parts of the target may have different effects on stability. In addition, an increase in temperature may cause differences not occurring at physiological temperature, therefore, Δ* T*_m_ may not accurately predict the efficacy of drug candidate under physiological conditions.

Fluorescence, differential scanning calorimetry (DSC) and circular dichroism (CD) spectroscopy are often used tools for monitoring thermal stability^3–8^. DSC is the recognized gold standard technique for measuring protein thermal stability, but it cannot give similar information about the exact structural changes in the studied protein as CD. Even valuable tools to study proteins, unfortunately, both methods share common limitations such low sensitivity and limited throughput^9,10^. This makes these methods material and time consuming especially when larger ligand panels are studied. To overcome these limitations, differential scanning fluorimetry (DSF) has become an increasingly practical and popular alternative to study protein thermal stability. DSF utilizes external dyes and thus it needs no target protein labeling, and assays can be executed employing relatively simple instrumentation. In DSF, many different external protein stability sensing dyes have been used and developed, but only few have gained wider popularity^11^. SYPRO Orange, which enables the direct use of qPCR equipment, is currently the most popular DSF dye. Independently of the exact probe structure and mechanism, all external probes target hydrophobic parts of the target proteins exposed during the protein unfolding, resulting in an increase in the fluorescence. However, as these probes sense their environment, dye structure and assay buffer selection may affect the assay functionality. In addition, properties of some assay buffer components itself, e.g., tris and histidine, are altered by heating^12^. These factors need to be considered to obtain reliable data using DSF^13,14^. To overcome these limitations, labelling of target proteins with fluorophore tags was recently introduced to DSF for better understanding of proteins unfolding in complex physiological systems^15–17^. While being a valuable technique, it cannot be universally applied since it needs to be customized for each specific protein being studied.

In addition to the use of external dyes, NanoDSF (nDSF) enables sensitive and precise method that determines protein unfolding by monitoring changes in the intrinsic fluorescence of the protein or by using fluorescently labeled protein^18^. In case of intrinsic protein fluorescence, denaturation of the protein causes change in the environmental surroundings of tyrosine and tryptophan amino acids leading to a shift in their fluorescence spectra and intensity, enabling also direct protein chemical stability monitoring^19^. In addition to nDSF, intrinsic Förster resonance energy transfer (iFRET), which is based on energy transfer from the tryptophan intrinsic fluorescence to a fluorescent labeled probe, can be used^20^. This technique, however, has been found problematic due to the high sensitivity of the tyrosine and tryptophan to the surrounding microenvironment, which might cause signal quenching or a spectral shift. Especially for chemical denaturation, addition of the chemical denaturant by itself might cause a shift in the fluorescence spectra, which is not actually related to the protein unfolding^18^.

Even TSA is often used, monitoring protein stability at physiological temperature may give better view about ligand binding and its effect on protein stability^21,22^. In TSA, heating causes proteins to acquire more kinetic energy, which breaks down weak hydrogen bonds and disrupts protein tertiary structure, leading to structural unfolding of protein. In case of chemical denaturation, the mechanism of protein unfolding differs depending on the denaturing agent^23^. Alcohols are often used for protein precipitation and denaturation, and they function by disrupting the protein tertiary structure by making hydrogen bonds with the protein side chains^24^. Urea, on the other hand, causes protein unfolding either directly through interaction with protein hydrophobic parts and water molecules, or indirectly through alteration of the solvent composition.^25–27^. In addition, low and high pH can cause protein denaturation, by causing protonation or deprotonation depending on isoelectric point (PI) of the protein and used pH^28^.

In case of isothermal chemical denaturation (ICD), mostly run in the presence of urea and guanidium chloride as denaturants, binding affinities can be simply obtained, which is not the case with TSA^21,29^. The downside in ICD is that these denaturants often require long incubation time to reach equilibrium in a denaturant titration, and high concentration of denaturant may also affect the affinity of the studied inhibitor. To overcome the limited throughput of ICD and relatively low sensitivity of both TSA and ICD, we have developed new member for the Protein-Probe family of techniques. The original Protein-Probe assay was based on protein stability sensing using a Eu^3+^-chelate peptide-probe, a highly polar glutamic-acid-rich peptide. This negatively charged probe has low interaction with the native protein compared to the denatured form, as it is believed that the main interaction of Eu^3+^-probe occurs through the protein hydrophobic core. However, the Protein-Probe method functionality rely on acidic pH, due to partial protonation of the peptide, and thus the assay must be performed in two steps, heating of the protein then addition of the detection solution, prior the monitoring of the time-resolved luminescence (TRL) signal. Even the method is highly sensitive, significantly improved sensitivity over the commercial reference methods, the need for low pH limits its use in single-step assay, typical for TSA and ICD^30–34^. Thus, we started to modify the system to enable protein thermal profiling at single-step and neutral pH, by changing the signal modulator to a more positive peptidic structure. However, by modifying only the modulator structure, we still end up two component system relying on electrostatic interactions, which might be more sensitive for unwanted responses in ICD. Thus, we developed here presented the single-peptide-probe for proteins stability studies, named as the FRET-Probe. In the FRET-Probe system, developed peptide-probe is labeled at both ends using Eu^3+^-chelate and Cy5 fluorophore. In the assays, the structure of the dual-labeled FRET-Probe changes upon binding to denatured protein, reducing the distance between labels. This provides an increase in time-resolved Förster resonance energy transfer (TR-FRET) signal with denatured protein, as low TR-FRET is monitored with the native or ligand stabilized protein. With the used model proteins, the FRET-Probe was found suitable for both TSA and ICD principles, enabling sensitive monitoring of protein–ligand interactions (PLIs) at nanomolar protein concentrations.

## Results and discussion

### Overview of the FRET-Probe structure and functionality

Stability of native proteins is strictly attributed to a narrow range of conditions of the solvent components and thermodynamic parameters^26^. Protein unfolding can occur due to many external stimuli, either physical like heat and radiation, or chemical like pH and organic compounds. Current methodologies to study protein unfolding under these varying denaturation conditions are unfortunately restricted by their limited sensitivity. Previously, we have introduced the Protein-Probe method, working under specific modulation conditions as an endpoint TSA. The Protein-Probe technique was developed to overcome the sensitivity limitation of the existing TSA methods, and we obtained approx. 100-fold improved sensitivity using nanomolar protein concentration levels. However, the endpoint assay protocol makes the assay inconvenient for TSA, and thus this technique was found to be more useful for the detection of protein aggregates and protease activity of native unmodified proteins^30,32^. Thus, we designed a modified Protein-Probe family member called the FRET-Probe to overcome limitations related to the use of two detection components and low pH modulation condition, enabling real-time stability monitoring at neutral pH.

**Figure 1:**
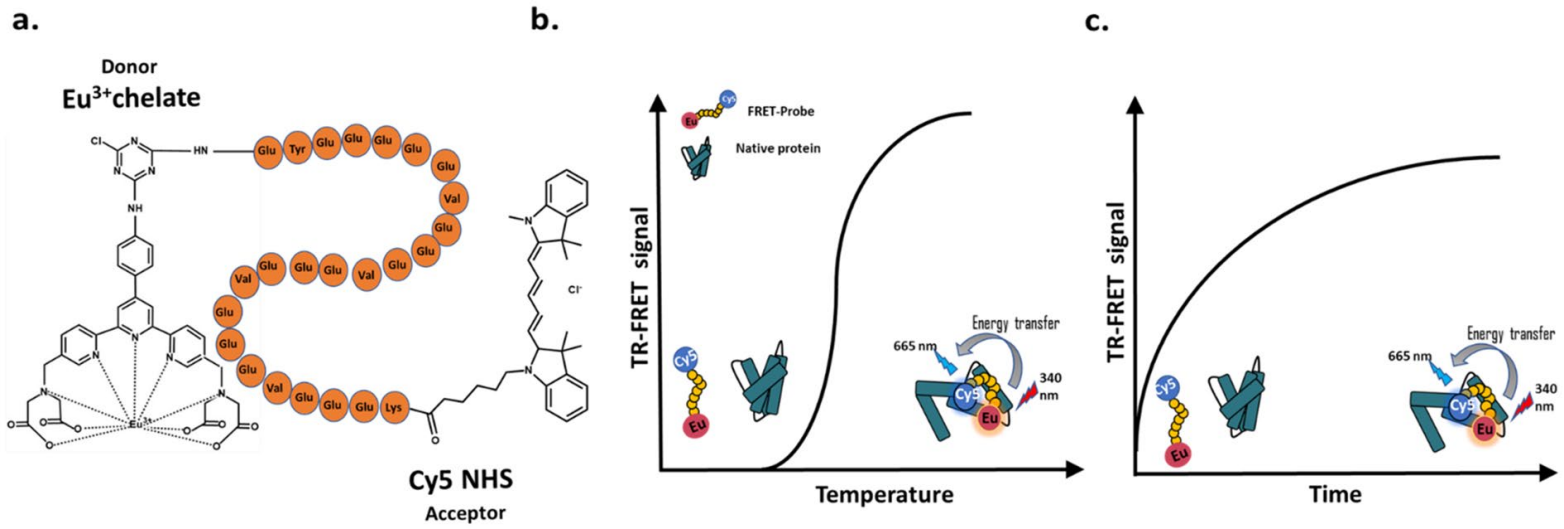
Structure and principle of the FRET-Probe. (**a**) The FRET-Probe is a peptide conjugated to Eu^3+^-chelate at the N-terminus and Cy5 at the C-terminus of a highly polar glutamic-acid-rich peptide sequence. Binding with the unfolded protein decreases the distance between the labels, allowing energy transfer to occur and thus increasing the TR-FRET signal monitored with heating in the TSA (**b**) or with longer incubation of the protein with the chemical denaturant in the ICD assay (**c**). In ICD assays, IC_50_ values are calculated at RT from the sigmoidal curve formed of the TR-FRET signal obtained in presence of a titration of the inhibitor concentration during the chemical denaturation of proteins at a specific time point.

The FRET-Probe consists of N-terminal Eu^3+^chelate donor and C-terminal Cy5-fluorophore as an acceptor conjugated to a peptide containing 24 amino acids (Fig. 1a). The peptide sequence of the FRET-Probe comprises four repeating units formed of three glutamic acids and one valine, with an additional three glutamic acids and one tyrosine at the N-terminus and three glutamic acids and one lysine at the C-terminus. By using two peptide conjugated labels, the system enables the use of a single detection component, unlike in other Protein-Probe-type assay platforms that have separate Eu^3+^-probe and Cy5-containing modulator. This additionally leads to monitoring of TR-FRET signal, unlike in other Protein-Probe-type assays, in which the Eu^3+^-chelate TRL-signal is monitored. Even the exact binding mechanism of the FRET-Probe, as also previously reported Eu^3+^-probe is still partly unknown, the interaction with the denatured protein increases the observed TR-FRET signal, as the signal with native protein is negligible. Both in the case of heat or chemically induced denaturation, binding of the FRET-Probe leads to a decrease in the donor and acceptor dye distance and increase in TR-FRET signal (Fig. 1). Interaction between the FRET-Probe and unfolded protein also protects the labels from their surroundings indicating that not only the probe sequence, but also fluorescent labels play a role in the detection of the denaturation event. As the FRET-Probe works independently of the denaturation method, we studied its functionality for PLIs in the ICD assay, previously not possible with the other Protein-Probe techniques (Fig. S1). As there is no TR-FRET applicable qPCR device, we systematically studied the FRET-Probe with a single denaturant concentration in a time-dependent manner, in a way enabling single concentration ligand library screening (Fig. 1c).

### FRET-Probe as a tool for protein thermal stability monitoring

**Figure 2:**
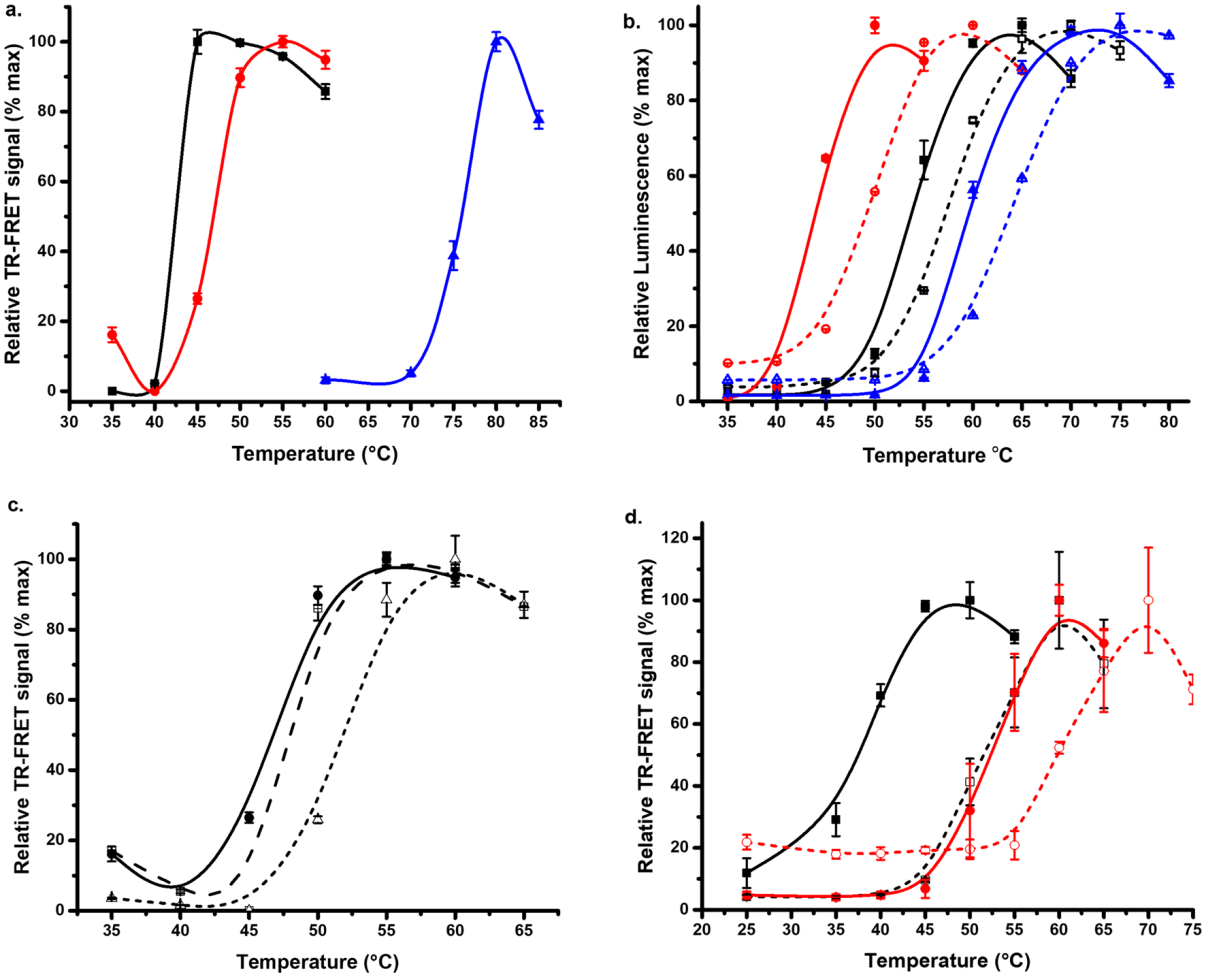
Protein thermal stability and protein ligand interaction (PLI) monitoring. (**a**) Thermal stability of SOS^cat^ (10 nM, black), MDH (25 nM, red), and trastuzumab (20 nM, blue) using the single-step FRET-Probe assay, *T*_m_ values were, 42.3 ± 1.6, 46.2 ± 0.4, and 75.4 ± 0.1 °C respectively. (**b**) Comparison of the FRET-Probe (solid) and Protein-Probe (dashed) assays for 150 nM KRAS^WT^ (black), KRAS^G13D^ (red), and KRAS^Q61R^ (blue), FRET-Probe *T*_*m*_ values were 53.6 ± 0.4, 44.7 ± 0.1 and 59.2 ± 0.3 °C, whereas Protein-Probe *T*_m_ values were, 57.2 ± 0.2, 49.9 ± 0.2 and 64.1 ± 0.19 °C respectively. (**c**) Thermal stability of 25 nM MDH (solid) with 5 (dashed) and 50 µM (dotted) NADH, *T*_*m*_ values were 46.7 ± 0.9, 47.9 ± 0.6 and 51.7 ± 0.2 °C respectively. (**d**) Thermal stability of 25 nM KRAS^G12C^ with (red) and without (black) 30 min preincubated adagrasib (250 nM), in the absence of Mg^2+^ (solid), *T*_*m*_ values were 38.5 ± 0.4 and 53.2 ± 1.2 °C respectively, whereas in presence (dashed) of Mg^2+^ (200 µM), *T*_*m*_ values were 53.5 ± 0.4 and 62.2 ± 0.9 °C respectively. Data represents mean ± SD (n = 3).

As the FRET-Probe is functionally and structurally distinct from our previous Eu^3+^-probe-based systems, we first validated it in a TSA format. Assays were performed utilizing three proteins, Son of Sevenless catalytic domain (SOS^cat^, 10 nM), trastuzumab (25 nM), and malate dehydrogenase (MDH, 20 nM) (Fig. 2a, Table S1). The assay was performed in a single step fashion, and data clearly showed the functionality of the FRET-Probe assay. *T*_m_ values measured for SOS^cat^, MDH and trastuzumab were 42.3 ± 1.6, 46.2 ± 0.4, and 75.4 ± 0.1 °C, respectively (Fig. 2a). These values were similar to the reported values, 45, 50 and 75 °C measured using Protein-Probe, fluorescence spectroscopy, and DSF, respectively^32,35,36^. All proteins gave high signal-to-background ratio and due to the atypical stepwise heating with 5 °C interval, a sharp transition curve was observed.

Next, we compared the FRET-Probe performance alongside the two-component and two-step Protein-Probe assay as both methods work in the same protein concentration range. The comparison was carried out using KRAS^WT^ and two mutants (KRAS^G13D^ and KRAS^Q61R^) as target proteins (Fig. 2b, Table S1). These mutants were selected due to their different intrinsic nucleotide exchange activity leading to different stability profiles^34^. *T*_*m*_ values of 57.2 ± 0.2, 49.9 ± 0.2 and 64.1 ± 0.2 °C were measured with the Protein-Probe assay, respectively. *T*_m_ values with the FRET-Probe assay, 53.6 ± 0.4, 44.7 ± 0.1 and 59.2 ± 0.3 °C, were downshifted, but the rank order of the *T*_m_ values was the same in both assays (Fig. 2b). KRAS^G13D^ was the least stable protein with Δ*T*_m_ 7.3 for Protein-Probe and 8.9 °C for FRET-Probe compared to KRAS^WT^, while KRAS^Q61R^ was the most stable with Δ*T*_m_ 6.9 for Protein-Probe and 5.6 °C for FRET-Probe (Fig. 2b). These small differences in the observed *T*_m_ values can be attributed to the varying assay conditions, as the assays are performed in different buffers and with different protocols. In Protein-Probe, KRAS is heated in 8 µL volume in which the concentration was 150 nM, but the detection is performed by adding 65 µL of detection, thus diluting the sample for the detection. On the other hand, the FRET-Probe method was performed in a 25 µL final volume using a single step protocol.

From these KRAS proteins we selected KRAS^Q61R^, as it was less protein and Mg^2+^ concentration dependent and tested it with two commercial DSF methods: GloMelt and SYPRO Orange (Fig. S2). This selection was made as it is known that *T*_m_ is affected by protein concentration. In addition, KRAS is affected by Mg^2+^ concentration, thus, protein *Tm* values cannot be easily compared with different techniques that were run at varying assay setup.^34,37^. With the commercial dye, 5 µM KRAS^Q61R^ was used, as the FRET-Probe assay was performed using 0.15 µM, still significantly higher than the lowest usable concentration for KRAS with the FRET-Probe. All methods efficiently measured the thermal stability of KRAS^Q61R^, having highly similar *T*_*m*_ values 59.5 ± 0.2, 61.2 ± 0.4 and 61.8 ± 0.3 °C using FRET-Probe, GloMelt, and SYPRO Orange, respectively (Fig. S2). Similar *T*_m_ values indicate that the FRET-Probe monitors the protein denaturation equally to GloMelt, and SYPRO Orange, but with increased sensitivity.

To further study the FRET-Probe, model PLI assays were next conducted for MDH with reduced nicotinamide adenine dinucleotide (NADH), and KRAS^G12C^ with its covalent inhibitor adagrasib^39,40^. To ensure that the results are solely attributable to the effect of the ligands, the FRET-Probe method was performed initially by incubating the protein and the ligand in plate, to enable interaction, before the FRET-Probe addition and the heating cycle. Additionally, each assay included a positive (protein only) and a negative (buffer only) control. It is known that many ligands stabilize native protein structure when bound, due to change of the unfolding-dissociation equilibrium^38,39^. As a first model, we chose MDH/NADH due to its low affinity interaction (K_D_ = 3.8 µM)^40^. Using 5 µM NADH concentration, MDH was only partially loaded with the ligand and thermal shift was moderate Δ* T*_m_ = 1.2 °C (Fig. 2c). By increasing the MDH loading to 50 µM NADH, MDH thermal shift was more pronounced Δ* T*_m_ = 5.0 °C. Thereafter, we studied high affinity interaction with KRAS^G12C^ and adagrasib. Adagrasib is known to interact with GDP-bound form of KRAS^G12C^ with nanomolar affinity, forming a covalent bond with the cysteine. Assay was performed in a buffer without and with 200 µM Mg^2+^, as Mg^2+^ is needed to preserve GDP loading, and using KRAS^G12V^ as a negative control^41^. For 25 nM KRAS, a typical Mg^2+^ induced effect on KRAS^G12V^ and KRAS^G12C^ stability was observed (Fig. 2d and S1). In the absence of Mg^2+^, *T*_m_ values of 38.3 ± 0.2 and 38.5 ± 0.4 °C were monitored, respectively. In the presence of Mg^2+^, KRAS^G12V^ and KRAS^G12C^ were both stabilized, and the *T*_m_ values of 53.2 ± 0.4 and 53.5 ± 0.4 °C were measured, respectively. As expected, 250 nM adagrasib did not have any ligand-induced stabilization for KRAS^G12V^ in presence or absence of Mg^2+^ (Fig. S3). However, significant KRAS^G12C^ stabilization was found with adagrasib, both in the absence (Δ* T*_m_ 14.7 °C) and presence (Δ* T*_m_ 8.7 °C) of Mg^2+^. Obtained data indicates that as low as 1.25 µM Mg^2+^, originated from KRAS storage buffer, is sufficient to preserve KRAS^G12C^ GDP-loading, and to induce stabilization of KRAS^G12C^ with adagrasib (Fig. 2d).

### Denaturation through protonation at low pH

As the FRET-Probe showed promising functionality in the TSA, we continued to study FRET-Probe in the ICD context. TSA requires heating and subsequent rapid or continuous reading during the heating cycle. As no real-time TR-FRET reader with temperature ramping exists, the interest for isothermal detection became obvious. Typically, ICD assays involve subjecting the target protein to a prolonged incubation with an increasing concentration of the chemical denaturant until equilibrium is achieved. This process can be accelerated by a mild isothermal heating, still preserving physiological or near physiological conditions^42,43^. Although ICD provides a distinct advantage over TSA, as it measures stability at ambient temperature, assay format with long incubation and multiple denaturant concentrations for each sample makes ICD somewhat impractical especially for PLI assays due to the lowered throughput compared to TSA^44,45^. Thus, we hypothesized that by combination of the nanomolar FRET-Probe sensitivity, high throughput properties of TSA, and positive aspects related to ICD, such as preserved protein structure and function^29,42,46^, reliable data can be obtained especially for PLIs. To this end, we developed a novel approach for ICD monitoring at room temperature (RT) by selecting a single denaturant condition and monitoring the denaturation over time (Fig. 1c and S1). The most crucial step was to select an appropriate denaturant with correct concentration, and thus, several known mild denaturants were scanned to study the real-time protein stability and interactions utilizing the FRET-Probe. In all of the ICD assays, we tracked the time-dependent TR-FRET signal at multiple intervals over a 150-min incubation period.

**Figure 3:**
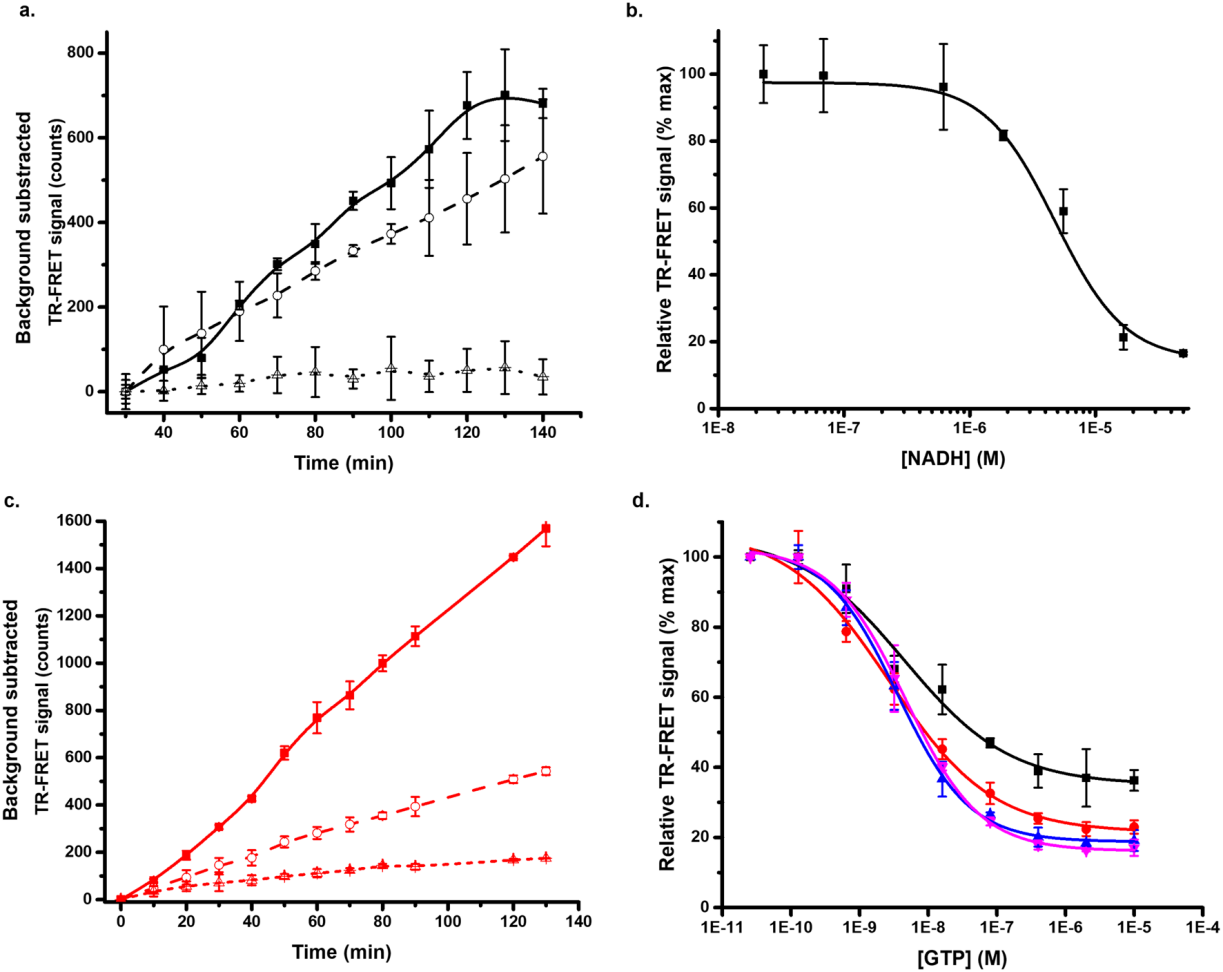
pH driven denaturation of KRAS^G12V^ and MDH. (**a**) Time-dependent pH 5 induced denaturation of 25 nM MDH (solid) with 5 (dashed) and 50 µM (dotted) NADH monitored at RT using the FRET-Probe. (**b**) NADH titration in the presence of constant MDH (25 nM) concentration in pH 5 denaturation buffer, EC_50_ value was 4.7 ± 0.8 µM. (**c**) pH 5 induced denaturation of 50 nM KRAS^G12V^ (solid) with 15 (dashed) and 2000 nM GTP (dotted) in a real-time FRET-Probe denaturation assay. (**d**) GTP titration in pH 5 denaturation buffer with constant KRAS^G12V^ (50 nM) concentration monitored at 30 (black), 60 (red), 90 (blue) and 120 (magenta) min time points, EC_50_ values were 4.5 ± 1.9, 2.6 ± 0.7, 3.5 ± 0.1, 4.6 ± 0.3 respectively. Data represents mean ± SD (n = 3).

Buffer pH is known to have a significant effect on protein stability, which needs to be additionally considered in all assay designs. In the original Protein-Probe platform, low pH was one of the key elements to modulate the assay function. Thus, low pH was first studied to evaluate the FRET-Probe ICD functionality^47^. For the TSA, neutral or slightly alkaline pH was used for our two main model proteins, MDH (25 nM) and KRAS (50 nM)^48,49^, as for the ICD assay, several acidic conditions were screened. Based on the preliminary testing, pH 5 was the mildest condition inducing denaturation of these model proteins in reasonable time window. At pH 5, MDH denaturates slowly with a 30-min lag-phase before the onset of the measurable denaturation process (Fig. 3a). When tested in PLI, using 5 and 50 µM NADH, a clear ligand-induced stabilization was observed showing also that lowered pH is compatible for the PLI monitoring. These results are comparable to those observed in TSA, indicating correct FRET-Probe function in the pH driven ICD setting (Fig. 3a). We further titrated MDH with several NADH concentrations and measured the signals multiple times at different time points. Based on the results obtained after 90 min incubation, we obtained an EC_50_ value of 4.7 ± 0.8 µM, which is well in line with that reported previously (Fig. 3b)^40^.

KRAS (50 nM) was shown to be more sensitive to acidic pH in comparison to MDH, as there was no lag phase when assayed at pH 5 (Fig. 3c). To test another PLI assay, KRAS^G12V^ was tested with guanosine-5'-triphosphate (GTP) as a ligand. GTP is essential for KRAS^G12V^ stabilization as it keeps KRAS^G12V^ in the nucleotide loaded state, and its affinity is very high even though the interaction in non-covalent. Addition of excess of GTP clearly stabilizes KRAS^G12V^, by preventing apo-KRAS formation. Surprisingly, our results showed that KRAS^G12V^ was stabilized already at nanomolar GTP concentration, much lower than previously measured with TSA (Fig. 3c)^34^. To confirm that the reduction in signal is related to GTP binding induced stabilization, a similar test was performed with ATP. As expected, TR-FRET signal level for the protein alone was in the same range as with ATP, even up to 10 µM concentration, confirming that GTP binding was the stabilizing factor (Fig. 3c and S4). To further study the PLI with GTP, TR-FRET signals for GTP titration were monitored at multiple time points. Longer incubation time increased the observed S/B ratio from 2.7 to 5.9, but no major change in the EC_50_ values of 4.5 ± 1.9, 2.6 ± 0.7, 3.5 ± 0.1 and 4.6 ± 0.3 nM, was observed at the four studied time points 30, 60, 90, and 120 min, respectively (Fig. 3d).

MDH and KRAS^G12V^ exhibit different molecular weights and clearly also their stability differs (Fig. 2a and Fig. 3)^50,51^. However, these both proteins are quite unstable and thus pH 5 was already destabilizing the structure enough to enable assays with the FRET-Probe. This is not the case with all proteins, some highly stable in acidic conditions, and thus pH as a denaturant is not expected to be suitable for all proteins. In addition, pH might also affect the negatively charged FRET-Probe or its labels, even Eu^3+^-chelate and Cy5 are rather stable. Thus, the method is expected to function only in rather mildly acidic conditions, which are unable to unfold stable proteins.

### Denaturation by altering protein tertiary structure using alcohols

**Figure 4:**
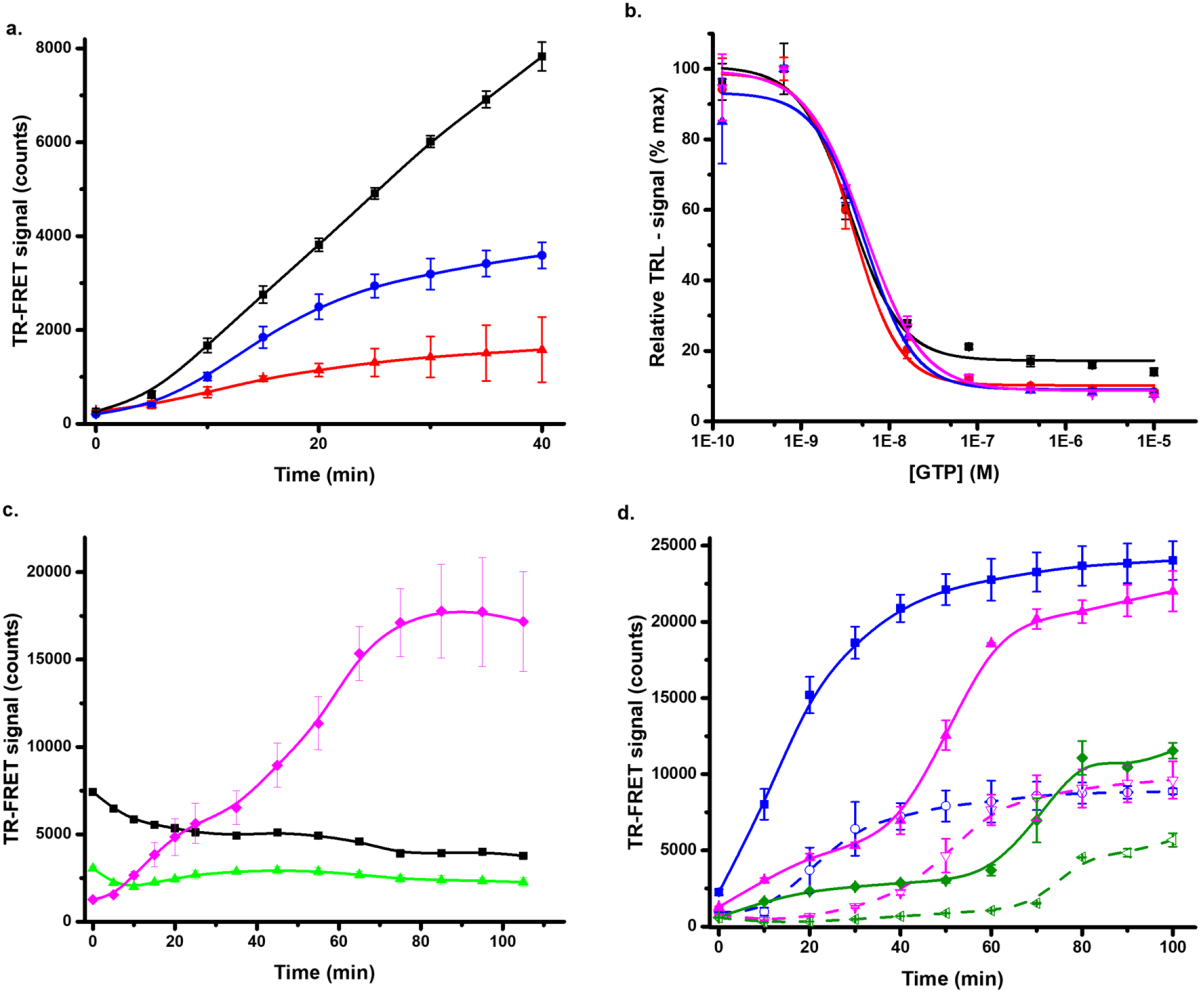
1-propanol induced denaturation of KRAS^G12V^ and MDH monitored with the FRET-Probe at RT. (**a**) Destabilization of KRAS^G12V^ (50 nM) was observed with 20 (red), 25 (blue) and 30% (black) 1-propanol at RT. (**b**) GTP titration in 20% 1-propanol monitored at 30 (black), 60 (red), 90 (blue) and 120 (magenta) min, EC_50_ values were 3.6 ± 0.5, 3.8 ± 0.4, 5.2 ± 1.2 and 5.2 ± 0.7 respectively. (**c**) MDH 25 nM (black) denaturation with 20% (green) and 40% (magenta) 1-propanol. (**d**) Propanol denaturation of 25 nM MDH (solid) with 30 (blue), 40 (magenta) and 50% (green) in presence of NADH 50 µM (dashed), higher S/B was obtained at lower alcohol concentration. Data represents mean ± SD (n = 3).

Alcohols such as ethanol, methanol, propanol, and butanol have been proposed for chemical denaturation of proteins due to their high content of hydrocarbon and water miscibility, facilitating the unfolding of native structure at a relatively low dose^24,52^. We selected ethanol and 1-propanol for FRET-Probe based protein denaturation testing at RT. In case of KRAS^G12V^ (50 nM), a relatively high alcohol concentration was required for full denaturation, 1-propanol being more efficient than ethanol. Interestingly, 1-propanol also gave an improved concentration dependency in comparison to ethanol for KRAS^G12V^ denaturation when 20, 25 and 30% alcohol were compared (Fig. 4a, Fig. S5a). Data for 1-propanol is shown at optimal 40 min time point as it demonstrates the best separation among the different alcohol concentrations tested. In 20% and 25% 1-propanol, maximal signal was reached after 40 min incubation, whereas in the 30% 1-propanol, maximal signal was monitored at 60 min (Fig. 4a). EDTA is expected to destabilize KRAS by chelating the Mg^2+^ required to preserve nucleotide loading, therefore, the assay was conducted in the presence of EDTA (0.2 mM) to examine its impact on denaturation rate (Fig. S5, Fig. S6a). With both alcohols, EDTA accelerated the denaturation rate and increased the overall TR-FRET signal compared to assays without EDTA. However, EDTA had basically no effect on optimal ethanol concentration (30%) (Fig. S5), but there was a significant effect on the optimal 1-propanol concentration, 20% in the absence and 4% in the presence of EDTA (Fig. S6a). When EDTA was tested with adagrasib and KRAS^G12C^, IC_50_ values obtained after 60 min incubation were similar with 30% ethanol, 4% 1-propanol + EDTA, and 20% propanol after 60 min incubation, 18.1 ± 0.1, 26.6 ± 2.3, and 19.2 ± 1.8 nM, respectively (Fig. S6b). To test the EDTA effect on a non-covalent KRAS binder, GTP titration was conducted in the presence of 20% 1-propanol (Fig. 4b). Similar to the pH denaturation assay, we titrated GTP and measured TR-FRET signals at different time points from 30 to 120 min. We observed that the monitoring duration during alcohol denaturation had no effect on the EC_50_ values of GTP (3.5–5.2 nM), and only moderate effect on the S/B ratio.

MDH was more tolerant to pH than KRAS^G12V^, and therefore, the alcohol denaturation experiments were tested with a wider range of alcohol concentrations. In a preliminary test, ~ 50% ethanol was sufficient for MDH denaturation. We demonstrated with KRAS^G12V^ that ethanol is a milder denaturant than 1-propanol, and thus, we proceeded to test 1-propanol concentrations below 40%. MDH did not show any response at 20%, but denaturation was seen at 40% concentration (Fig. 4c). Based on this data we further tested 1-propanol at concentrations between 30 and 50% in a PLI assay with NADH. Interestingly, MDH was denatured at 30% 1-propanol with higher S/B than that obtained at 40% and 50% concentration of 1-propanol. This decrease in the S/B ratio might be attributed to protein aggregation or interference with the FRET-Probe binding at high alcohol concentration (Fig. 4d)^53^. Moreover, NADH stabilizing effect remained consistent across all tested alcohol concentrations with EC_50_ values at 60 min incubation of 29–38 µM (Fig. 4d). These results indicate that the high 1-propanol concentration does not affect the FRET-Probe binding, at least by affecting the EC_50_ values monitored.

Both tested alcohols are FRET-Probe compatible, but as in case of pH, target protein stability affects the needed optimal concentration. Based on the results, stable proteins cannot be efficiently used as over 50% alcohol concentrations are not easily usable, even no interference to FRET-Probe functionality was seen. In addition, evaporation starts to play a role when high alcohol concentration and long incubation times are used. Mild heating could induce alcohol effects, but this would increase the evaporation related variation. Interestingly, alcohol as a denaturant seems to increase the observed TR-FRET signal those observed in TSA, as lowered pH had opposite effect.

### Denaturation by altering protein hydration using urea

**Figure 5:**
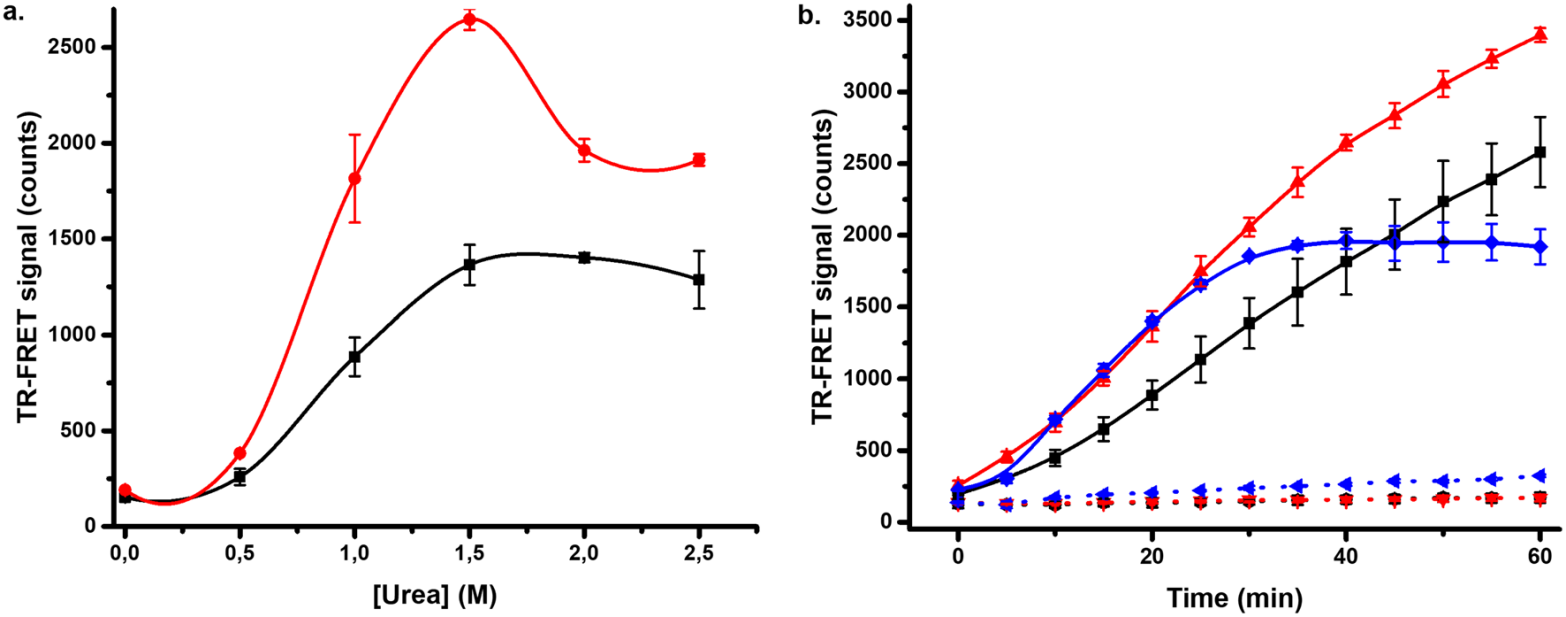
Urea induced denaturation of KRAS^G12V^. (**a**) 0–2.5 M urea was titrated with 50 nM KRAS^G12V^ and monitored after 20 (black) and 40 min (red) of incubation at RT to examine the impact of incubation time on the optimal urea concentration. (**b**) The time dependence of KRAS^G12V^ 50 nM (solid) and stabilizing effect of 1 µM GTP (dashed) was observed with 1 (black), 1.5 (red) and 2 M (blue) urea, when KRAS^G12V^ denaturation was monitored with and without GTP after 60 min incubation in 1 and 1.5 M urea, S/B ratios of 15.1 and 20.3 were recorded, respectively. Data represents mean ± SD (n = 3).

Urea is one of the most used chemical denaturants, and it was selected as a third chemical denaturant to study the FRET-Probe^54^. Again, MDH and KRAS^G12V^ were first investigated to assess the optimum urea concentration (0–5 M). Based on the preliminary tests, MDH denaturation was not measurable, as urea concentrations above 3 M had a negative effect on the FRET-Probe function (data not shown). Based on previous reports, MDH is relatively stable in urea, and denatures at 5–6 M urea concentration^55,56^. Therefore, we focused on KRAS^G12V^ showing a clear response at low 1–3 M urea concentration (data not shown). When the urea concentration was studied further, incubation time was found to have an influence on the optimal urea concentration (Fig. 5a). This can be highlighted in an assay with 50 nM KRAS^G12V^ monitored in the absence and presence of GTP (1 µM) and using 1, 1.5, and 2 M urea. The rank order of different urea concentrations was changed over time, as in the highest urea concentration (2 M) the observed TR-FRET signal saturated after 30 min, much faster than with the other two lower concentrations (Fig. 5b). No saturation was reached with either 1 or 1.5 M urea during the 60 min incubation. As in the previous tests with other denaturants, KRAS^G12V^ stability also increased in the urea assay in the presence of GTP (Fig. 5b). When KRAS^G12V^ denaturation reactions with and without GTP were compared at optimal 60 min time point, high S/B ratios of 15.1 and 20.3 were monitored with 1.5 and 2 M urea, respectively (Fig. 5b). Urea is typically used at high 5 to 8 M solutions and it is known that some proteins can tolerate these even these conditions for several hour^26,55–57^. However, we selected not to study protein denaturation with extended period of time, but rather keep the assay time short. Urea denaturation can also be combined with heating, lowering the concentration demand, but to keep assay simple, we performed all assays at room temperature.

### Comparison of denaturants using covalent KRAS^G12C^ inhibitors

Due to the different functionalities of the different denaturants with different proteins, it is important to optimize the assay for the selected target. Our results consistently demonstrated that alcohol denaturation occurs in concentration dependent manner and is dependent on the used alcohol. Also with mildly acidic pH, chosen model proteins were efficiently denatured in few hours. On the other hand, urea denaturation with KRAS showed consistent results and high S/B ratio with low variation, but on the other hand, MDH could not be denatured. As KRAS^G12V^ denaturation was visible with all denaturants, we next compared the conditions with several KRAS^G12C^ covalent inhibitors.

**Figure 6:**
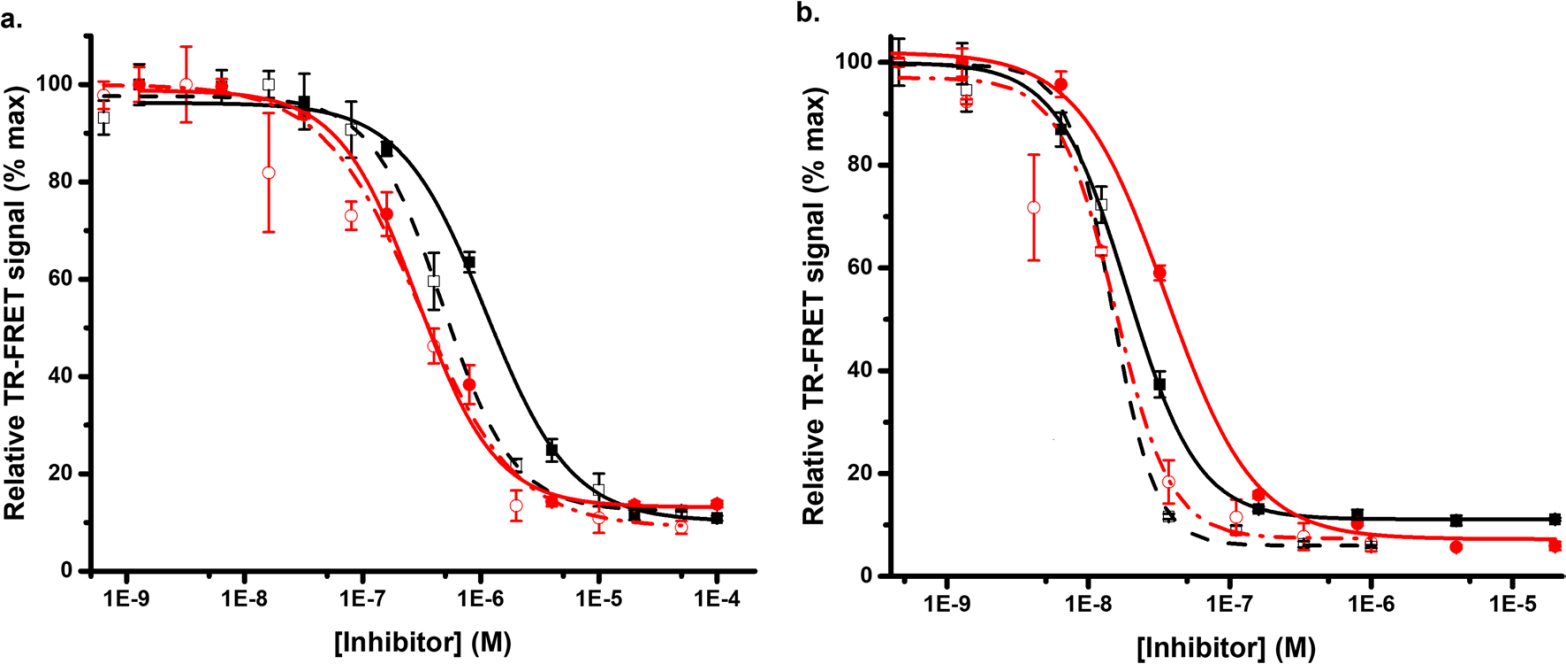
Urea denaturation monitoring with different KRAS^G12C^ covalent inhibitors using the FRET-Probe. (**a**) ARS853 (black) and ARS1620 (red) inhibitor titration for 50 nM KRAS^G12C^ with urea (dashed) in comparison to QRET nucleotide exchange assay data (solid). The IC_50_ values after 60 min with urea and QRET nucleotide exchange for ARS853 were 1104 ± 126 and 353 ± 50 nM, and for ARS1620 were 289 ± 55 and 240 ± 36 respectively. (**b**) Adagrasib (black) and sotorasib (red) inhibition of 50 nM KRAS^G12C^ with urea (dashed) in comparison to nucleotide exchange (solid). IC_50_ with urea and nucleotide exchange assay for adagrasib were 14.5 ± 1.1 and 18.9 ± 0.7 nM, and for sotorasib, 15.4 ± 1.2 and 35 ± 7.1 nM, respectively. Data represents mean ± SD (n = 3).

For the inhibitor testing, two early generation KRAS^G12C^ inhibitors (ARS1620 and ARS853) having high nM to low µM binding affinity were selected^58,59^, together with two recently FDA approved nanomolar binders AMG510 (sotorasib) and MRTX849 (adagrasib)^60^. These covalent inhibitors bind to the GDP-loaded KRAS^G12C^ at the switch II pocket, interfering SOS binding and nucleotide exchange keeping RAS in its inactive form^61^. We analyzed these four inhibitors first by using SOS^cat^ catalyzed nucleotide exchange assay, which is based on quenching resonance energy transfer (QRET). In the assay, Eu^3+^-GTP binds to KRAS^G12C^ giving high TRL-signal in the absence of inhibitor, whereas TRL-signal is low in the presence of nucleotide exchange inhibitor^34,62,63^, All inhibitors showed expected stabilizing effect on KRAS^G12C^ structure and blocking of KRAS^G12C^ nucleotide exchange in the control experiments (Fig. 6, Fig. S7). The IC_50_ values with QRET nucleotide exchange for ARS853 and ARS1620 were 353 ± 50 nM, and 240 ± 36, respectively (Fig. 6a), and for adagrasib and sotorasib were 18.9 ± 0.7 nM, and 35.0 ± 7.1 nM, respectively (Fig. 6b).

Thereafter, we tested ARS853 and ARS1620 using pH 5 buffer for denaturation. IC_50_ values were calculated after 60 min incubation at RT and those were 1017 ± 383 and 123 ± 35 nM for ARS853 and ARS1620, respectively (Fig. S7a). Sotorasib and adagrasib were also tested under the same conditions, and IC_50_ values of 25.0 ± 3.3 and 25.0 ± 4.6 nM were observed, respectively (data not shown). IC_50_ values are protein concentration dependent in nature, and thus, inhibitors with binding affinity below half of the protein concentration will not yield accurate IC_50_ value, but rather monitors protein concentration. In these experiments, KRAS^G12C^ concentration was 50 nM, and thus, IC_50_ values observed might not be completely accurate. However, these values are highly similar to those reported earlier^60^, but this highlights the importance of maintaining a low protein concentration in the assay, a distinctive aspect of our FRET-Probe methodology. Next, ARS1620 and ARS853 titration was conducted using 15–25% 1-propanol. We observed that the selected 1-propanol concentration influenced the monitored IC_50_ value calculated after 60 min incubation at RT (Fig. S7b). IC_50_ values for ARS853 and ARS1620 were 312 ± 23, 197 ± 62 and 1279 ± 98 nM and 40.1 ± 1.3, 54.0 ± 2.7, and 414 ± 8 nM in 15, 20, and 25% 1-propanol, respectively. The data clearly shows that the change in protein structure upon exposure to alcohol, is primarily dependent on the concentration of alcohol, but also that the used condition must be carefully selected, and values obtained in varying conditions might not be directly comparable^52^.

Next, urea-induced denaturation was tested for KRAS^G12C^ with ARS853 or ARS1620 using the same 60 min incubation time as with other denaturants. The IC_50_ values with urea for ARS853 and ARS1620 were 1104 ± 126 and 289 ± 55, respectively (Fig. 6a). The data with urea is in line with those observed with pH 5 ICD and QRET nucleotide exchange, and interestingly, data obtained with 25% 1-propanol is also comparable. However, lower isopropanol concentration had a tendency to lower the obtained IC_50_ values obtained. When adagrasib and sotorasib were monitored with urea, similar highly comparable results were measured with the FRET-Probe in urea and pH 5 ICD assays, and in the nucleotide exchange, IC_50_ with urea were 14.5 ± 1.1 and 15.4 ± 1.2 for adagrasib and sotorasib, respectively (Fig. 6b).

In this study, we introduce the FRET-Probe method enabling denaturation studies both using heating and chemical denaturants. We selected two distinct model proteins, KRAS and MDH exhibiting contrasting characteristics with regards to size and structure, and used these proteins to analyze functionality of different denaturation conditions. Following a comprehensive study of the model proteins with three chemical denaturants, each having a unique mechanism of action, our data showed that MDH, was more tolerant to all three denaturants than KRAS, even both proteins are relatively unstable. MDH cannot be denatured in used urea concentrations in a used incubation time at RT. In contrast, KRAS was measurable with all denaturants and its stability can be easily further adjusted by using varying Mg^2+^ concentrations or EDTA. As an opposite of MDH, urea denaturation was preferred over alcohols and pH with KRAS, in terms of S/B, reproducibility, and kinetics. As the FRET-Probe method using single denaturant concentration at RT is mainly targeted to PLI analysis, ligand binding studies were performed to compare the functionality. Importantly, IC_50_ values obtained using varying ICD conditions were comparable and also in line with values in literature and reference methods. As an exception, low alcohol concentrations had a tendency to provide lower IC_50_ values with KRAS^G12C^ inhibitors than other methods. Therefore, it is clear that method selection is dependent on the studied protein. KRAS^G12C^ inhibitor studies also showed the importance of assay sensitivity, as the FRET-Probe could monitor IC_50_ values also for nanomolar binders. This is not possible with other label-free stability assays, as their limited sensitivity forces the use of micromolar level target protein concentrations.

## Methods

The detailed list of materials and instrumentation, synthesis, and purification of proteins and Eu^3+^ conjugates are presented in the (supplementary data).

### Thermal shift assays

All FRET-Probe TSA were conducted in triplicates using a single-step protocol, by adding the sample protein in 5 µL and FRET-Probe (0.5 nM) in 20 µL volumes. All concentrations are reported in a 25 µL final volume. Assays were performed in an assay buffer containing 10 mM HEPES (pH 7.5) supplemented with 0.001% (v/v) Triton X-100. Trastuzumab (20 nM), SOS^cat^ (10 nM), MDH (25 nM), and KRAS (25–150 nM) were all assayed between 35–90 °C using 5 °C steps and heating the sample 2 min at each step prior TR-FRET measurement. Additionally, MDH was assayed in the presence of NADH (0–50 µM) and KRAS^G12C^ with adagrasib (0–0.25 µM) in a buffer with or without additional MgCl_2_ (0.2 mM).

TSA control assays were performed using the Protein-Probe. Assay in conducted using two step protocol, in which protein concentration was calculated in 8 µL volume used for sample heating. Samples were heated 3 min at each temperature followed by addition of 65 µL of Protein-Probe detection solution and TRL-signal monitoring. KRAS^WT^, KRAS^G13D^ and KRAS^Q61R^ (150 nM) were added in sample buffer (10 mM HEPES, (v/v) 0.001% Triton X-100) and Protein-Probe detection solution containing citrate–phosphate buffer (7.7 mM Na_2_HPO_4_ and 6.1 mM citric acid, pH 4) supplemented with 0.01% (v/v) Triton X-100, 3.5 µM HIDC, and 1 nM Eu^3+^-probe.

SYPRO Orange and GloMelt assays were performed by a one-step protocol using 5 µM KRAS^Q61R^ in buffer (20 mM HEPES (pH 7.5), 100 mM NaCl, 5% glycerol). Samples (16 µL) and SYPRO Orange/GloMelt solution (4 µL) were combined prior to the first heating step in a white 96-well plate (BioRad). The samples were incubated for 1.5 min at each temperature, followed by fluorescence signal measurement every 2 °C. SYPRO Orange was used at 5 × (stock 5000 ×) and GloMelt at 1 × final concentration (stock 200x). Signals were monitored using 460 and 485 nm excitation and 510 and 590 nm emission wavelengths for SYPRO Orange and GloMelt, respectively.

### FRET-Probe isothermal chemical denaturation assays

All isothermal chemical denaturation (ICD) assays were conducted in triplicates at RT using a single-step protocol with 25 µL final volume. Sample protein and ligand were added in 5 µL in a buffer (10 mM HEPES (pH 7.5), 10 mM NaCl, (v/v) 0.01% Brij 30) without denaturant. FRET-Probe (0.5 nM) was added to the assay buffer supplemented with chemical denaturant, urea (0–5 M), ethanol (0–30%) or 1-propanol (0–50%). Additionally, 0.2 mM EDTA was used in combination with alcohols. Acid denaturation was performed in citrate phosphate buffer (10.3 mM Na_2_HPO_4_ and 4.85 mM citric acid, pH 5) supplemented with 0.01% (v/v) Brij 30.

In all ICD protein assays, time-resolved Förster resonance energy transfer (TR-FRET) signal was monitored kinetically at multiple time points during 150 min incubation. In all assays with protein binding ligands, 30 min preincubation prior denaturant addition was performed. In acid induced denaturation assay, GTP (0–2 µM) and NADH (0–50 µM) were titrated with KRAS^G12V^ and MDH, respectively. 1-propanol (30–50%) ICD assays were also performed for MDH in the presence of NADH (0–50 µM) and (20%) for KRAS^G12V^ using GTP (0–10 µM). Ethanol (30%) and 1-propanol (4 & 20%) were tested with KRAS^G12C^ using adagrasib (2 µM) single concentration or in a titration (0–10 µM). ARS1620 and ARS853 (0–50 µM) or adagrasib and sotorasib (0–2 µM) titrations were performed with KRAS^G12C^ using pH 5, 1-propanol (15–25%) and urea (1 M) as a denaturant. Additionally, urea was tested with 1 µM GTP with KRAS^G12V^.

### Nucleotide exchange assay

The nucleotide exchange was performed by incubating KRAS^G12C^ (50 nM) with the covalent inhibitor, sotorasib and adagrasib (0–1 µM) or ARS1620 and ARS853 (0–50 µM) for 30 min in 10 µL volume. Thereafter, 5 µL of detection solution (3 µM Q14, 10 nM Eu^3+^-GTP) was added and reaction was initiated with 10 nM SOS^cat^. TRL-signals were monitored multiple times during 60 min incubation. Assays were performed in a buffer containing 20 mM HEPES (pH 7.5) 1 mM MgCl_2_, 10 mM NaCl, 0.01% Triton X-100. The assay was performed using triplicate reactions.

### Data analysis

In all assays, the signal-to-background ratio (S/B) was calculated as µ_max_/µ_min_ and coefficient of variation (CV%) (σ/µ) *100, where µ is the mean value and σ is the standard deviation. *T*_*m*_ values, EC_50_, and IC_50_ values were obtained using standard sigmoidal fitting functions with fitting equation, y = A2 + (A1-A2)/(1 + (x/ × 0)^p). Data were analyzed using Origin 2016 software (Origin Lab, Northampton, MA).

## Conclusion

Protein denaturation by heating (TSA) is the most often used method to study protein stability and especially PLIs. Although ICD is often proposed to provide biologically more relevant and accurate information, fluorescence-based TSA assays are simpler and faster to perform especially with a panel of ligands^21,22^. This study demonstrates that the novel FRET-Probe assay design is high throughput compatible, and both heat and temperature can be equally used for protein denaturation. The FRET-Probe assay allows assays with nanomolar sensitivity independently of the denaturation condition, and also PLI studies using single-step protocol. This is a remarkable improvement to our previously developed Protein-Probe family of assays and compared to current commercial methods typically working at micromolar protein concentrations. Assay sensitivity not only saves materials, but also enables accurate binding studies also for nanomolar binders. This makes the FRET-Probe assay design a powerful tool especially for high throughput ligand screening without a need for special instrument but using a standard plate reader under isothermal condition. However, the exact binding mechanism of the FRET-Probe is unknown, and the universality of the method for different types of proteins is yet to be unraveled. It is likely that not all proteins can be studied at low nanomolar concentrations and that mild denaturation conditions used in the study are not equally suitable for all proteins. However, as ICD can be easily combined with mild heating, some of the more stable proteins might also be studied with the ICD method. It also remains unknown if harsher denaturation conditions could be used with an increased FRET-Probe concentration or with varying buffer composition. For this, more research to understand the FRET-Probe binding mechanism is needed.

### Supplementary Information

Supplementary Information.

## Data Availability

Data for this article will be made available from the corresponding author upon reasonable request.
