# Supplementary material for: Isothermal chemical denaturation assay for monitoring protein stability and inhibitor interactions
Source: Sci Rep. 2023 Nov 16;13:20066. doi: 10.1038/s41598-023-46720-w (PMC10654576; doi:10.1038/s41598-023-46720-w)
Supplement: Supplementary file 1 — Supplementary Information. [file 41598_2023_46720_MOESM1_ESM.docx]

**Appendix A. Supporting informations**

**Isothermal chemical denaturation assay for monitoring protein stability and inhibitors interactions**

Randa Mahran^1*^, Niklas Vello^1^, Anita Komulainen^1^, Morteza Malakoutikhah^1^, Harri Härmä^1^, Kari Kopra^1^

*****Corresponding author email address: randa.r.mahran@utu.fi

**Table of contents**

1. **Materials and instrumentation**

**Table S1.** Proteins and their characteristics**.**

1. **Supporting results**

**Figure S1**. FRET-Probe principle for detection of PLI

**Figure S2.** KRAS^Q61R^ thermal stability with different methods.

**Figure S3**. FRET-Probe assay for KRAS^G12V^ thermal stability and protein-ligand interaction (PLI) monitoring

**Figure S4.** ATP interaction with KRAS^G12V^ in pH driven denaturation.

**Figure S5.** Ethanol and EDTA induced denaturation for KRAS^G12V.^

**Figure S6.** Comparison of 1-propanol and ethanol induced KRAS^G12C^ denaturation with and without EDTA and adagrasib.

**Figure S7**. Comparison of different 1-propanol concentrations and pH 5 denaturation for KRAS^G12C^.

1. **Supporting references**

**1. Materials and instrumentation**

*E. coli* expression, and purification of SOS^cat^, KRAS^WT^, and KRAS mutants (KRAS^G12C^, KRAS^G12V^, KRAS^G13D^, and KRAS^Q61R^) are described elsewhere and proteins were a kind gift from Leidos Biomedical Research, Inc., Frederick National Laboratory for Cancer Research [1,2]. KRAS inhibitors, adagrasib was purchased from (Divbio Science Europe, Netherlands), ARS853 was from (Immuno Diagnostic Oy, Finland) & ARS1620 was obtained from (Cayman chemical, Michigan, USA). Eu^3+^-GTP and Protein-Probe labeling using the 9-dentate chelate, {2,2',2",2'"-{[4'-(4'"-isothiocyanatophenyl)-2,2',6',2"-terpyridine-6,6"-diyl]bis(methylene-nitrilo)}tetrakisacetate)} europium(III) according the manufacturer’s instructions (QRET Technologies, Turku, Finland) and as described before [1,3–5].

For FRET-Probe, peptide (H2N‐EYEEEEEVEEEVEEEVEEEVEEEK(Cy5) was obtained from (Pepmic Co., Ltd., Suzhou, China), and was conjugated to 9-dentate chelate, {2,2',2",2'"-{[4'-(4'"-dichlorotriazine)-2,2',6',2"-terpyridine-6,6"-diyl]bis(methylene-nitrilo)}tetrakisacetate)} europium(III) according to manufacturer’s instructions ((QRET Technologies, Turku, Finland), in the same way as described for the Protein-Probe. 1-propanol was acquired from (Fisher Scientific, USA). 1,1',3,3,3',3'-Hexamethylindodicarbocyanine iodide (HIDC), Malate dehydrogenase (MDH), trastuzumab, reduced nicotinamide adenine dinucleotide (NADH), guanosine-5'-triphosphate (GTP), urea and all solvents and buffer components were purchased from Sigma-Aldrich (St. Louis, MO, USA). FRET-Probe assays with heating were conducted in black 348-well PCR plates (4titude, Germany), and Protein-Probe control assays in black Framestar 96-well plates from (4titude). SYPRO Orange and GloMelt assays were performed in white 96-well plates (BioRad). Chemical denaturation assays were performed in black and nucleotide exchange in white 384-well low volume plates (Corning, USA).

All measurements were performed using a Spark 20M from Tecan Life Sciences (Männedorf, Switzerland). Thermal heating was performed with a PTC-100 Programmable Thermal Controller (MJ Research, Inc., Watertown, MA). Time-resolved luminescence (TRL) signals were monitored using 340/620 nm excitation/emission wavelengths, and 800 µs and 400 µs delay and integration times, respectively. In all FRET-Probe assays, signal was monitored using 340 nm excitation and 665 nm emission wavelengths and with 50 µs delay and 200 µs integration times. All purifications were performed using reverse-phase liquid chromatography Dionex ultimate 3000 LC system from Thermo Fischer Scientific, Dionex, and Ascentis RP-amide C18 column (Sigma-Aldrich, Supelco Analytical) as previously described [5,6]. The specifications for all proteins tested in the study are listed in Table 1.

**Table S1. Protein and their characteristics.**

| Protein | Function | Mw (kDa) | Structure | Isoelectric point  (pI) | *T_m_*  (°C) | Ref |
| --- | --- | --- | --- | --- | --- | --- |
| SOS^cat^ | a guanine exchange factor that activates RAS proteins | 57 | Catalytic domain (564-1048) | * | 45 | [7] |
| MDH | Malate oxidizing enzyme | 70 | Dimer | 6.1-6.4 | 45-50 | [7,8] |
| Trastuzumab | HER2 binding monoclonal antibody, brand name Herceptin | 148 | IgG1 | 8 -8.9 | 80-83 | [9,10] |
| KRAS^WT^ | Small GTPase | 21 | Monomer (2-188) | 6.1-6.4 | 53-74 | [11–13] |
| KRAS^G12V^ | Mutated constantly active small GTPase | 21 | Monomer (2-188), glycine 12 to valine point mutation | 6.1-6.4 | 52-60 | [14–16] |
| KRAS^G12C^ | Mutated constantly active small GTPase | 21 | Monomer (2-188), glycine 12 to cysteine point mutation | 6.1-6.4 | 53-62 | [2] |
| KRAS^G13D^ | Mutated constantly active small GTPase | 21 | Monomer (2-188), glycine 13 to aspartic acid point mutation | 6.1-6.4 | 43-48 | [2] |
| KRAS^Q61R^ | Mutated constantly active small GTPase | 21 | Monomer (2-188), glutamine 61 to arginine point mutation | 6.1-6.4 | 51-67 | [2] |

- The data was not available

**2. Supporting Results**

**
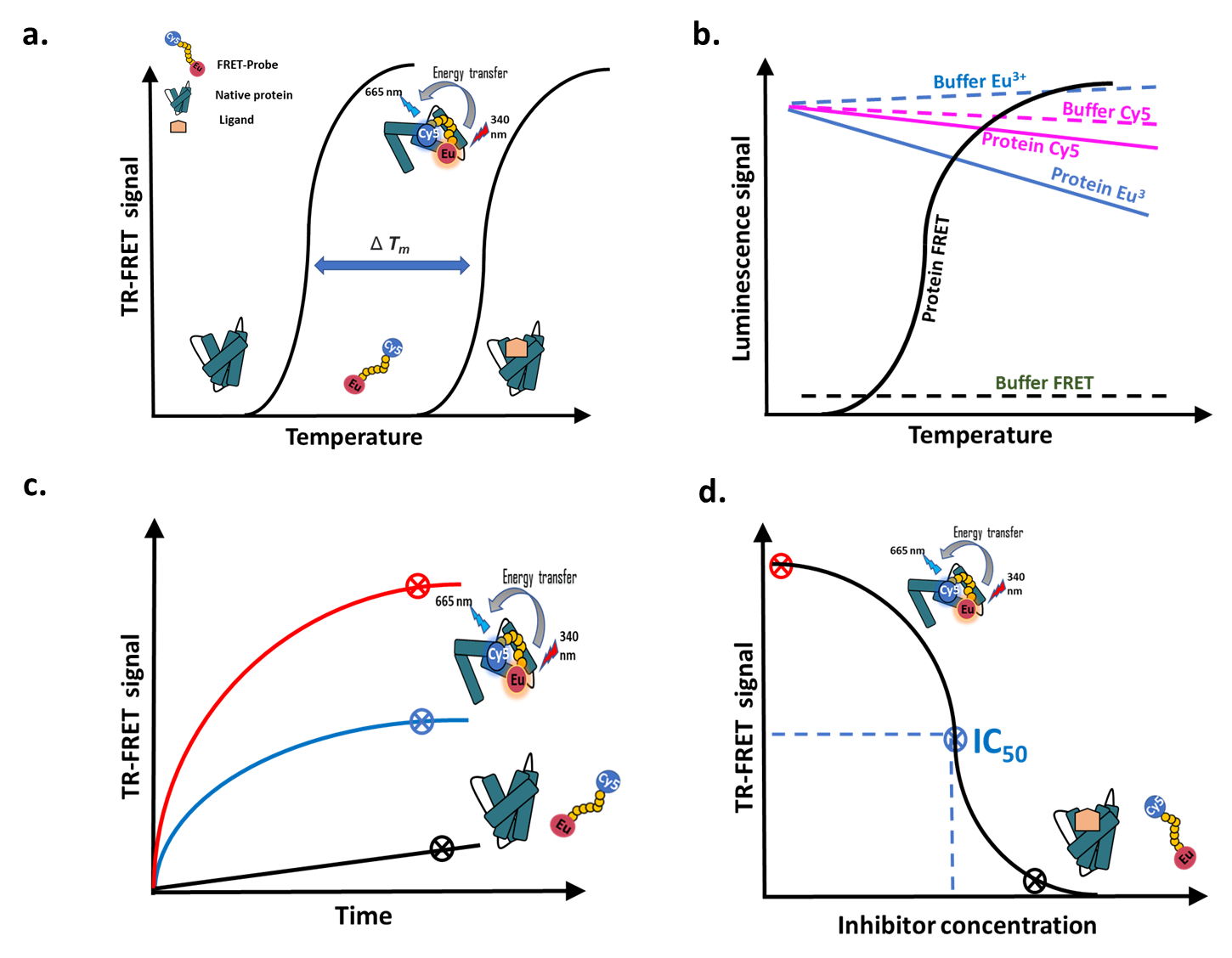
**

**Figure S1**. FRET-Probe principle for the detection of ligand induced target protein heat or chemical denaturation. **a.** In the thermal shift assay (TSA), low time-resolved Förster resonance energy transfer (TR-FRET) signal is monitored at low temperatures prior proteins denaturation induced FRET-Probe binding, increasing the observed TR-FRET signal. In the presence of target protein bound ligand, protein preserves its stability at higher temperatures in comparison to native protein, thus shifting the *T*_m_ value of the protein. This shift is referred as *ΔT_m_*, indicating this stability increase. **b.** In TSA assay with the FRET-Probe, TR-FRET signal in the presence of the protein (black, solid) increases due to the structural unfolding at higher temperatures, as in the absence of protein, low TR-FRET signal (black, dashed) is monitored at all cases. The increase in TR-FRET causes a decrease in Eu^3+^-chelate time-resolved luminescence (TRL) signal in the presence of the protein (blue), due to the energy transfer to Cy5, of which fluorescence (magenta) stays unchanged in all cases. **c.** In the ICD assay, protein denaturation is monitored in a time dependent manner in the presence of mild chemical denaturant. In the absence of ligand, protein denaturation increases the monitored TR-FRET signal (red), as bound ligand reduces the signal in concentration dependent manner by reducing the protein denaturation (blue and black). **d.** From the ICD results, IC_50_ values can be calculated by blotting the observed TR-FRET signals at the selected time point against the ligand concentration. High TR-FRET signal is observed at low ligand concentration and decrease in signal at high ligand concentration, forming a typical sigmoidal curve shape.

**Figure S2**. KRAS^Q61R^ thermal stability with different methods. Thermal stability of KRAS^Q61R^ 0.15 µM with FRET-Probe (black), 5 µM with GloMelt (red) and 5 µM with SYPRO Orange (blue) was monitored, all methods showed relatively similar *T_m_* values; 59.5 ± 0.2, 61.2 ± 0.4 and 61.8 ± 0.3 °C respectively. FRET-Probe method had much higher sensitivity, and the assay was performed in 33-fold lower concentration. Data represents mean ± SD (n=3).

**Figure S3.** KRAS^G12V^ thermal stability and protein-ligand interaction (PLI) monitoring using the FRET-probe. Thermal stability of 25 nM KRAS^G12V^ was monitored without (red) or with (black) 200 µM MgCl_2_, in the absence (solid) or presence (dashed) of adagrasib (250 nM). Adagrasib showed no thermal stability change with KRAS^G12V^, indicating no interaction. Mg^2+^ had clear KRAS^G12V^ stabilizing effect (53.2 ± 0.4 °C) in comparison to assay without Mg^2+^ (38.3 ± 0.2 °C). Data represents mean ± SD (n=3).

**Figure S4.** ATP interaction with KRAS^G12V^ in pH driven denaturation. pH 5 induced denaturation of 50 nM KRAS^G12V^ was monitored in absence (black) and presence of 2 (red) and 10 (blue) µM ATP, showing no change of signal level in presence of ATP. Data represents mean ± SD (n=3).

**
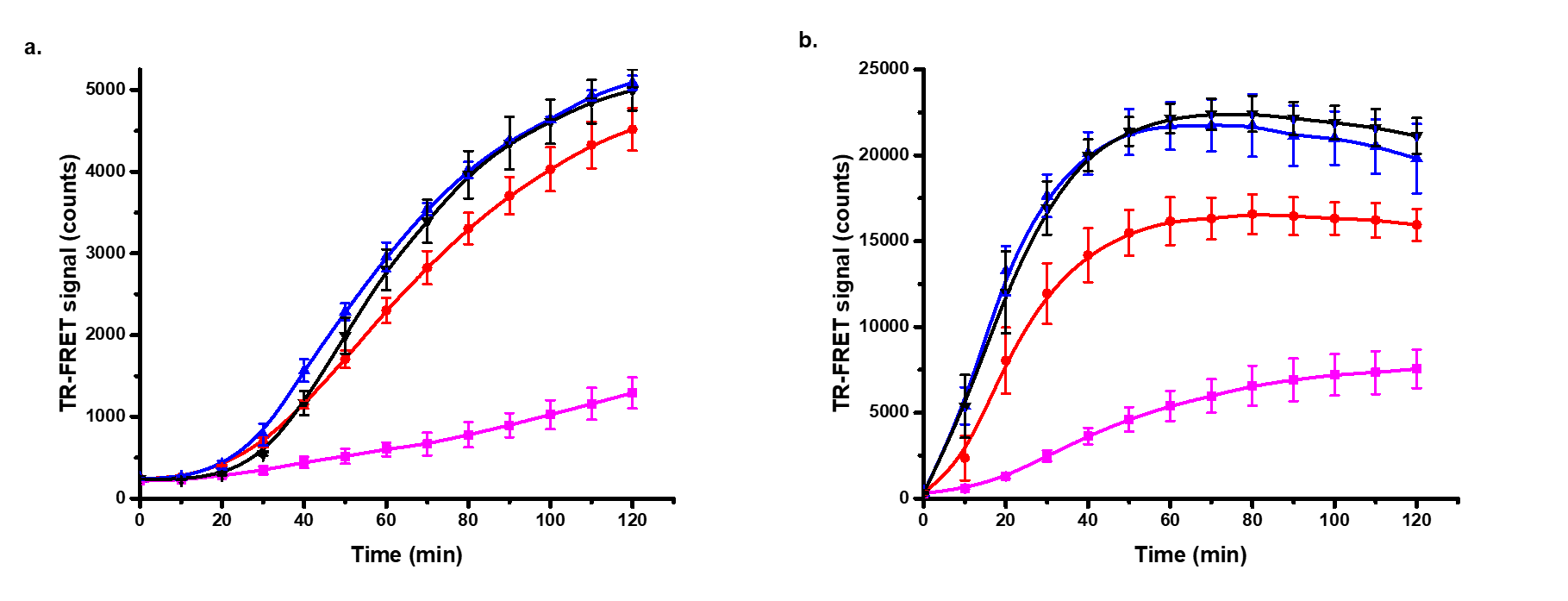
**

**Figure S5.** Ethanol and EDTA induced denaturation for KRAS^G12V^. KRAS^G12V^ (50 nM) (magenta) denaturation assay without **a.** and with **b.** 0.2 mM EDTA, with 20% (red), 25% (blue), and 30% (black) ethanol. There was no clear change in denaturation performed with 20-30% ethanol, either in signal level or speed. EDTA has a synergistic effect to the ethanol denaturation leading to reduced denaturation time to 60 min. Data represents mean ± SD (n=3).


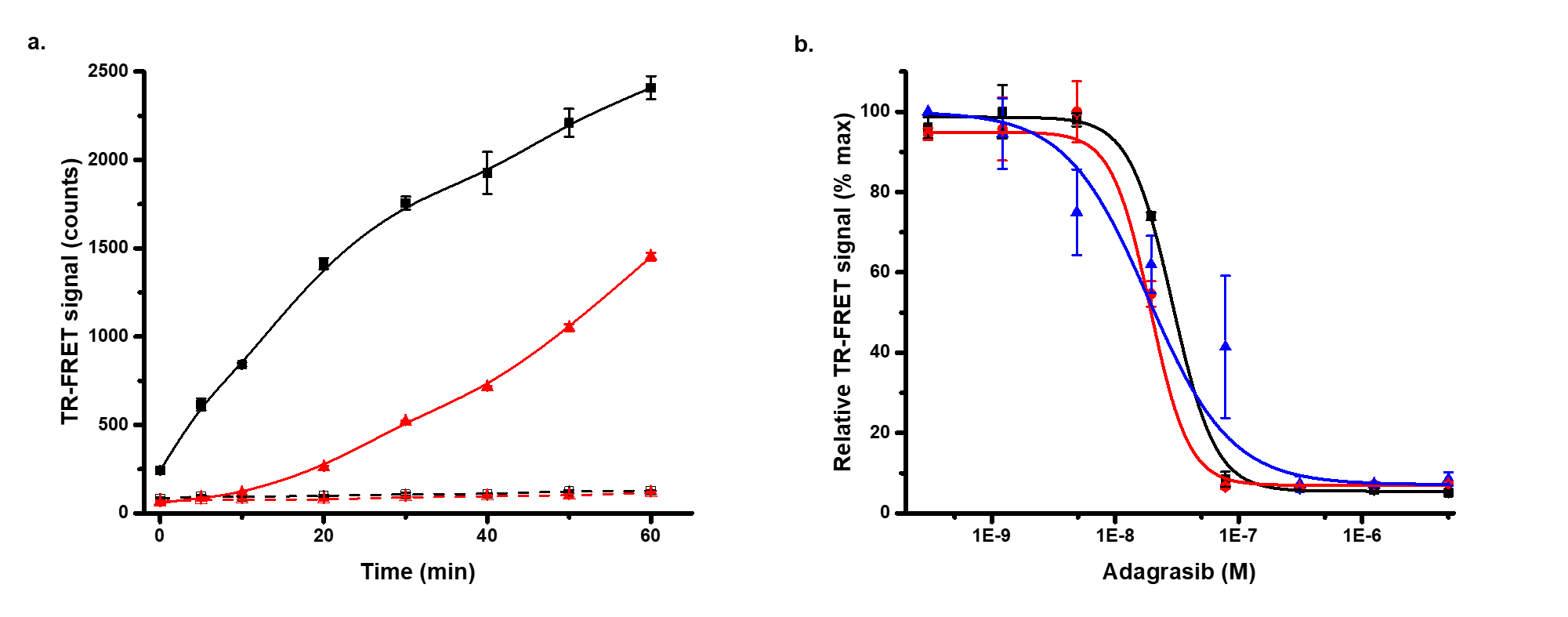


**Figure S6.** Comparison of 1-propanol and ethanol induced KRAS^G12C^ denaturation with and without EDTA. **a.** Denaturation of 50 nM KRAS^G12C^ without (solid) and with (dashed) 2 µM adagrasib 4% 1-propanol/ 0.2 mM EDTA (black), and 20% 1-propanol (red). In case of KRAS^G12C^, EDTA significantly reduced the 1-propanol concentration required for denaturation as Mg^2+^ crucial for KRAS stability is chelated. **b.** Adagrasib inhibitor titration with KRAS^G12C^ (50 nM) in 30% ethanol/0.2 mM EDTA (blue), 4% 1-propanol/ 0.2 mM EDTA (black), and 20% 1-propanol (red). IC_50_ values obtained in these conditions were all similar, 18.1± 0.1, 26.6 ± 2.3, and 19.2 ± 1.8 nM, respectively. Data represents mean ± SD (n=3).

**
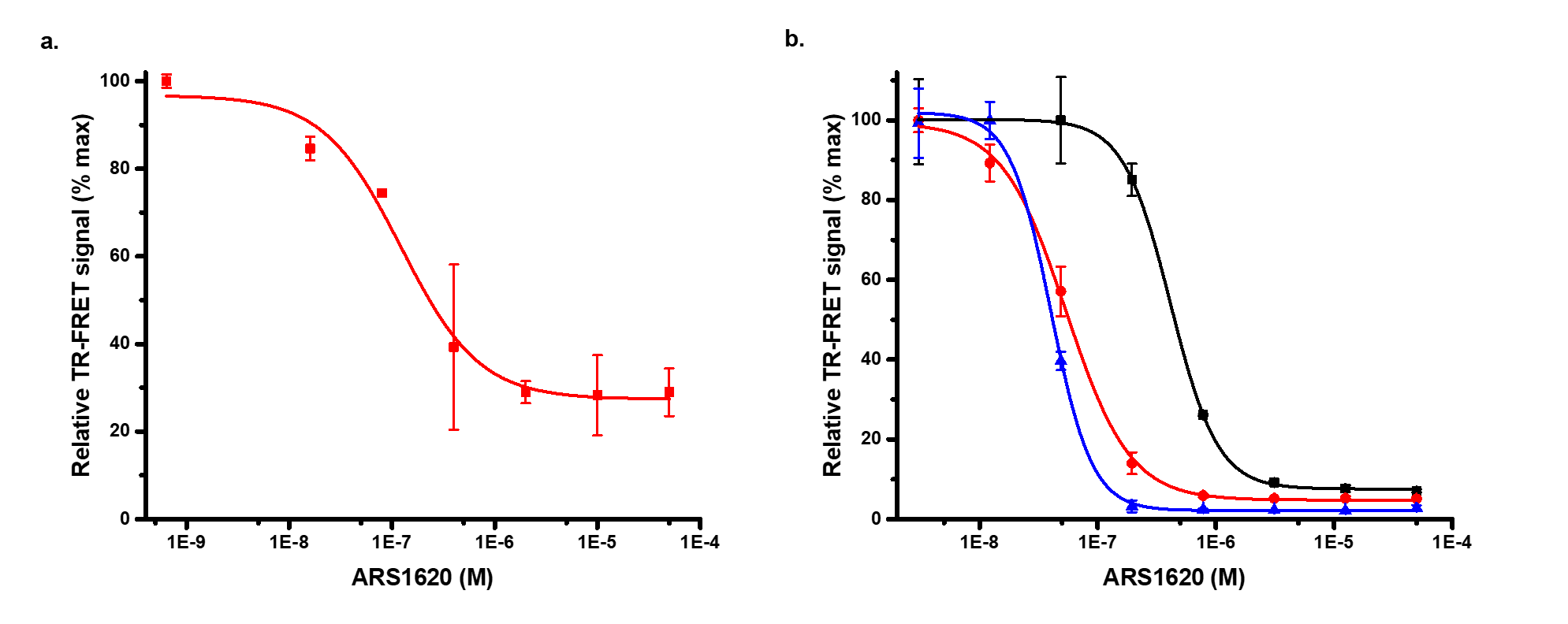
**

**Figure S7.** KRAS^G12C^ denaturation with different 1-propanol concentrations and in pH 5. **a.** ARS1620 inhibitor titration with KRAS^G12C^ (50 nM) in pH 5, EC_50_ value calculated at 60 min was 122 ± 34 nM, which correlated with the expected value. **b.** ARS1620 induced KRAS^G12C^ (50 nM) stabilization in 15% (blue), 20% (red) and 25% (black) 1-propanol. 1-propanol drastically changed the observed EC_50_ values, which were 40.1 ± 1.3, 54.0 ± 2.7, and 414 ± 8 nM in the presence of 15, 20, and 25% 1-propanol, respectively. Data represents mean ± SD (n=3).

**3. Supporting references**

1. Kopra, K. *et al.* Homogeneous dual-parametric-coupled assay for simultaneous nucleotide exchange and kras/raf-rbd interaction monitoring. *Anal Chem* **92,** 4971 (2020).
2. Kopra, K. *et al.* Thermal shift assay for small GTPase stability screening: evaluation and suitability. *Int J Mol Sci* **23,** 7095 (2022).
3. Kopra, K. *et al.* A Homogeneous Quenching Resonance Energy Transfer Assay for the Kinetic Analysis of the GTPase Nucleotide Exchange Reaction. *Anal Bioanal Chem* **406,** 4147–4156 (2014).
4. Syrjänpää, M., Vuorinen, E., Kulmala, S., Wang, Q., Härmä, H., & Kopra, K. QTR-FRET: Efficient Background Reduction Technology in Time-Resolved Förster Resonance Energy Transfer Assays. *Anal Chim Acta*, **1092,** 93–101(2019).
5. Vuorinen, E. *et al.* Sensitive label-free thermal stability assay for protein denaturation and protein-ligand interaction studies. *Anal Chem* **92,** 3512–3516 (2020).
6. Valtonen, S. *et al.* Nanomolar protein–protein interaction monitoring with a label-free protein-probe technique. *Anal Chem* **92,** 15781–15788 (2020).
7. Vuorinen, E. *et al.* Protease substrate‐independent universal assay for monitoring digestion of native unmodified proteins. *Int J Mol Sci* **22,** 6362 (2021).
8. Lourenço, E. C. Synthesis of new enzyme stabilisers inspired by compatible solutes of hyperthermophilic microorganisms (Doctoral dissertation, Universidade NOVA de Lisboa (Portugal)) (2013).
9. Khawli, L.A. *et al.* Charge Variants in IgG1 Isolation, Characterization, in Vitro Binding Properties and Pharmacokinetics in Rats. *MAbs* **2,** 613–624 (2010).
10. Sert, F. *et al.* Temperature and PH-Dependent Behaviors of MAb Drugs: A Case Study for Trastuzumab. *Sci Pharm* **90,** 21 (2022).
11. Kistler, S.K. Investigating the Role of Post-Translational Modifications in the Core RAS GTPase Domain (Doctoral dissertation, University of North Carolina (USA)) (2019).
12. Xu, K. *et al.* Small Molecule KRAS Agonist for Mutant KRAS Cancer Therapy. *Mol Cancer* **18,** 1–16 (2019).
13. Yin, G. *et al.* A KRAS GTPase K104Q Mutant Retains Downstream Signaling by Offsetting Defects in Regulation. *J Biol Chem* ***292*,** 4446 (2017).
14. Wright, K.M. *et al.* Hydrophobic Interactions Dominate the Recognition of a KRAS G12V Neoantigen. *Nat. Commun* ***14*,** 1–20 (2023).
15. Janssen, K. *et al*. Exploiting the Intrinsic Misfolding Propensity of the KRAS Oncoprotein. *Proc Natl Acad Sci U S A* ***120*,** e2214921120, (2023).
16. Monzon, F. A., Ogino, S., Hammond, M. E. H., Halling, K. C., Bloom, K. J., & Nikiforova, M. N. The Role of KRAS Mutation Testing in the Management of Patients with Metastatic Colorectal Cancer. *Arch Pathol Lab Med* ***133*,** 1600–1606 (2009).
